# Temperature Calibration Using Machine Learning Algorithms for Flexible Temperature Sensors

**DOI:** 10.3390/s25185932

**Published:** 2025-09-22

**Authors:** Ui-Jin Kim, Ju-Hun Ahn, Ji-Han Lee, Chang-Yull Lee

**Affiliations:** 1 Department of Aerospace Engineering and the Program in Aerospace Systems Convergence, Inha University, Incheon 21999, Republic of Korea; kuijin@inha.edu (U.-J.K.); ljihan@inha.edu (J.-H.L.); 2 Institute of Advanced Technologies for LEO Space Economy, Inha University, Incheon 21999, Republic of Korea; jhahn@ssml.re.kr; 3 Center for Aerospace Research, Inha University, Incheon 21999, Republic of Korea

**Keywords:** flexible temperature sensor, EHD inkjet printing, deep neural network, long short-term memory

## Abstract

Thermal imbalance can cause significant stress in large-scale structures such as bridges and buildings, negatively impacting their structural health. To assist in the structural health monitoring systems that analyze these thermal effects, a flexible temperature sensor was fabricated using EHD inkjet printing. However, the reliability of such printed sensors is challenged by complex dynamic hysteresis under rapid thermal changes. To address this, an LSTM calibration model was developed and trained exclusively on quasi-static data across the 20–70 °C temperature range, where it achieved a low prediction error, a 33.563% improvement over a conventional polynomial regression. More importantly, when tested on unseen dynamic data, this statically trained model demonstrated superior generalization, reducing the RMSE from 12.451 °C for the polynomial model to 4.899 °C. These results suggest that data-driven approaches like LSTM can be a highly effective solution for ensuring the reliability of flexible sensors in real-world SHM applications.

## 1. Introduction

Thermal imbalance in structures causes thermal stress, which negatively affects structural health. Local thermal stress is likely to occur in structures with large volumes and complex cross-sections, such as aircraft, bridges, and ships. These structures are often composed of materials with different thermal expansion coefficients, such as steel bars, concrete, and carbon composites. When the cross-sectional area is large, the distance from the heat source can be significant and can increase deformation and stress due to thermal imbalance. This phenomenon can reduce the lifespan and safety of structures. For these reasons, there is growing necessity for machine learning-based structural health monitoring using thermal analysis and sensor data. Among these structures, research is actively being conducted to analyze the thermal imbalance that occurs in composite structures such as bridges and concrete beams, and to propose a structural health monitoring system using deformation and time-series data [1,2]. Peng et al. [3] attached thermocouples and strain gauges to concrete-filled steel tubular structures. Based on the acquired data, they derived temperature gradients and strain curves at various locations and compared with actual measurements via finite element modeling. Alcover et al. [4] analyzed the deformation occurring on the Great Belt Bridge in Denmark with temperature load and traffic load data to perform structural health monitoring using regression equations. Furthermore, a study reported that the fatigue lifespan of composite material connections is influenced by temperature [5].

Temperature is a factor that has a significant impact on the lifespan and health of structures. The importance of structural health monitoring through precise temperature measurement is being emphasized accordingly. For this reason, it is essential to develop flexible sensor elements that can be attached to complex cross-sectional structures or irregular structures with curvature [6]. Inkjet printing is attracting attention as one of the key technologies for developing such flexible sensors [7]. Electrohydrodynamic (EHD) inkjet printing is receiving a lot of attention as a method for fabricating flexible temperature sensors [8]. EHD inkjet printing applies high voltage to a nozzle to generate an electric field, which interacts with the surface tension to print conductive ink that is finer than the nozzle diameter. Various flexible sensor elements based on this technology are being researched, and Ag and Carbon-based inks are mainly used to fabricate flexible temperature sensors [9]. Ahn et al. [10] developed ink for EHD inkjet printing by combining Ag nanoparticles with diethylene glycol monobutyl ether acetate and α-terpineol and used it to study printing parameters and fabricate a patch-type lattice sensor for heat detection. He et al. [11] used EHD inkjet printing to fabricate a high-resolution, micro-scale temperature sensor with a size of 450 μm × 450 μm. Kamal et al. [12] analyzed the reproducibility of the fabricated temperature sensor and compared the performance changes during additive printing. Lungulescu et al. [13] fabricated temperature sensors with carbon black-graphite and evaluated their positive temperature coefficient (PTC) properties performance.

Deep Neural Network (DNN) is a representative machine learning method that predicts values based on data. Long Short-Term Memory (LSTM) is a method that uses time-series data to predict values. Both methods apply weights to the given data and work by finding the optimal gradient through multiple layers to improve prediction performance [14,15,16,17]. Such deep learning models are attracting attention in the field of structural health monitoring, using various variables and sensors and attracting attention for their ability to effectively calibrate nonlinear patterns in sensor signal calibration [18,19]. Especially for sensors affected by cyclic thermal condition, DNN and LSTM methods that learn from time-series data are being used as effective tools for sensor calibration [20,21,22]. Ren et al. [23] used a neural network method to evaluate aspects of the ocean environment, such as temperature, through sensors and succeeded in reducing the mean absolute error (MAE) to 0.808% and 0.154 °C. Yu et al. [24] applied DNN methods to the calibration of silicon-based optical sensors to reduce errors compared with the Lorenz fitting algorithm. Shi et al. [25] used LSTM to detect abnormal thermal changes in aircraft systems.

While research on calibrating flexible sensors using machine learning is still in its infancy, this study proposes a robust data-driven model for this purpose. In this paper, a flexible temperature sensor was fabricated using EHD inkjet printing on a polyimide film, aiming to replace conventional sensors that are difficult to attach to irregular structures. The fabricated sensor exhibited a nonlinear resistance–temperature relationship, but the more critical challenge was the emergence of complex dynamic hysteresis under rapid thermal changes, which complicates accurate temperature determination in real-world scenarios. The goal of this study, therefore, was to investigate the potential for a single calibration model that can provide reliable predictions across a wide operational boundary, from quasi-static to dynamic conditions. MATLAB R2024b was used for experimental data processing, and TensorFlow 2.10.0 was used for machine learning methods. To improve the effectiveness of the sensor, machine learning algorithms, DNN and LSTM, were applied. These findings confirm that to build a comprehensive model covering this broad range of conditions, data-driven approaches are more effective than conventional methods like polynomial regression, which fail to address the complex errors in the dynamic regime. The most significant contribution is demonstrating that the LSTM model, trained exclusively on static data, shows smaller errors than the existing polynomial regression method when tested on unseen dynamic data, highlighting its superior generalization capabilities for real-world applications.

## 2. Properties of Flexible Temperature Sensors

### 2.1. Ink Manufacturing Process and EHD Inkjet Printing Parameters

**Figure 1:**
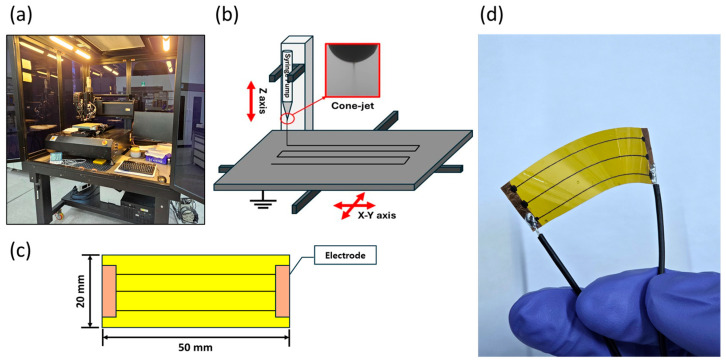
(**a**) EHD inkjet printing system, (**b**) schematic image, (**c**) sensor dimensions, and (**d**) verification of sensor flexibility.

This paper was conducted by fabricating sensors using an EHD inkjet printer (Enjet Inc., Suwon, Republic of Korea). EHD inkjet printing, which consists of the components shown in Figure 1, is a printing technique that applies high voltage to the ink ejection nozzle and grounds the substrate, generating an electric field that enables ink ejection through electrohydrodynamic interaction. When voltage is applied, the fluid surface of the ink is saturated by electrical interaction [26]. In this state, the ink is ejected alongside the electric field. The sensor was printed by the continuous inkjet method [27]. The CIJ method enables rapid printing by continuously applying voltage to eject ink.

**Figure 2:**
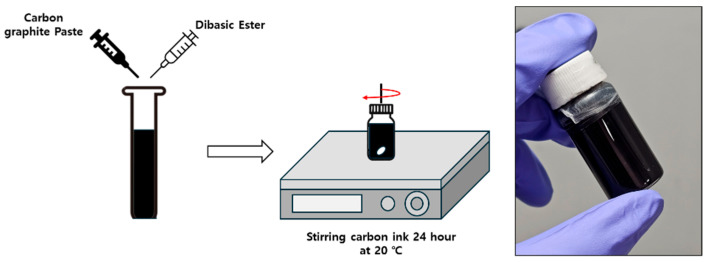
Process of making carbon-based ink for EHD inkjet printing.

The temperature sensor ink was made using carbon paste (Paron 920, Changsung Co., Incheon, Republic of Korea) and dibasic ester (DBE, Sigma-Aldrich, Saint Louis, MO, USA). Carbon-based inks composed of activated carbon and graphite have self-healing and deformation-resistant properties [28]. These properties help prevent sensor damage and performance degradation when attached to irregular surfaces. Figure 2 shows the manufacturing process for carbon-based ink. The carbon paste was sonicated for five minutes. Subsequently, the surfactant DBE was added, and the mixture was stirred for two minutes using a vortex mixer. Then, the ink was placed on a magnetic stirrer at 900 rpm and 20 °C for 24 h.

Unlike conventional inkjet printing, EHD inkjet printing applies high voltage in the kV range to the nozzle during the printing process. This enables the ejection of ink with a diameter thinner than the nozzle hole, but applying excessive voltage can cause sparks to occur in conductive ink and cause nozzle clogging. Therefore, it is essential to carefully adjust the printing parameters of the ink for stable EHD inkjet printing. Sensor fabrication was performed at 22.7 °C to align with room-temperature conditions commonly used in prior studies, and the relative humidity was maintained at 21% following the referenced work, a level reported to minimize hysteresis deviation [29,30]. Printing of the flexible temperature sensor was performed on a PI film.

**Figure 3:**
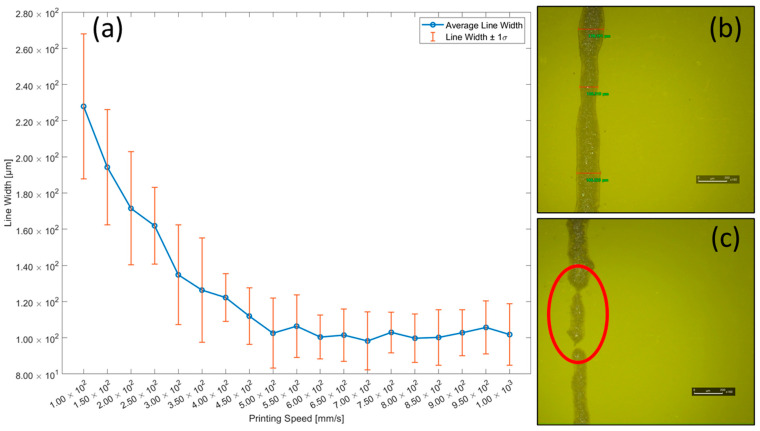
(**a**) Average line width differences according to printing speed [100–1000 mm/s], (**b**) 160× magnified image of a sensor sample printed at 250 mm/s, and (**c**) an image of a defective sensor line, with the point of discontinuity encircled in red.

In view of the enhanced sensor efficiency associated with reduced line width, the printing speed should be experimentally determined to obtain the most appropriate line width [8]. To determine optimal printing speed conditions, the sensor line width was measured at printing speeds ranging from 100 to 1000 mm/s in 50 mm/s increments, as shown in Figure 3. At each stage speed, three parallel lines were printed. Line width was measured at seven equidistant positions along each line, producing twenty-one measurements per speed. Width was reported as the means of these measurements. Dispersion was expressed as the standard deviation in Table 1. Tolerance was defined as mean ± σ. When the printing speed exceeded 300 mm/s, the reduction in line width with increasing speed became more gradual, indicating a smaller absolute slope of width versus speed than in the under 300 mm/s regime. From 400 mm/s onward, the standard deviation of the line width also decreased. The average line width printed at 100 mm/s was 227.905 ± 40.014 μm, which decreased to 161.917 ± 21.147 μm at 250 mm/s. Above 500 mm/s, there was almost no change in the line width, except for the minor variations caused by the individual differences between the sensors. Although increasing the printing speed above 500 mm/s reduced the line width, an increased occurrence of broken lines has been observed at speeds over 300 mm/s shown in Figure 3c. As a result, the optimal printing speed was determined to be 250 mm/s.

**Figure 4:**
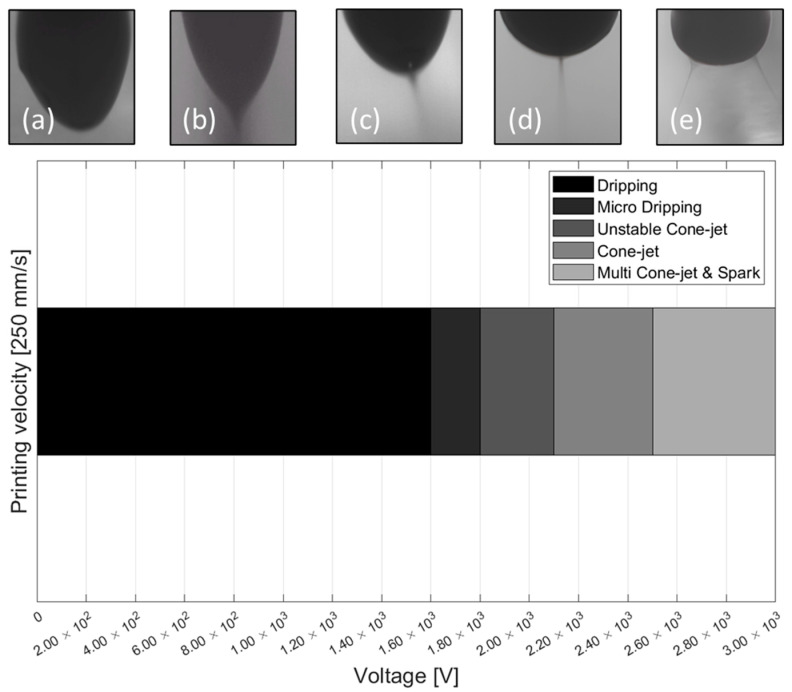
Supply voltage distribution map of EHD inkjet printing meniscus shapes: (**a**) dripping mode, (**b**) micro dripping mode, (**c**) unstable cone-jet mode, (**d**) cone-jet mode, and (**e**) multi cone-jet.

To obtain the cone-jet ejection mode, the voltage condition was increased by 0.05 kV at a fixed speed condition of 250 mm/s. Figure 4 shows the meniscus shape of the carbon ink and the voltage range. The printing results showed a dripping mode below 1.6 kV, and a micro-dripping mode was maintained until 1.8 kV. The unstable cone-jet mode began to appear at 1.85 kV, and the cone-jet mode was maintained from 2.1 kV to 2.5 kV. If a voltage higher than 2.5 kV was set, sparks and multi-cone-jet mode occurred, so the final print voltage was set to 2.3 kV. Table 2 shows the printing parameters of carbon ink.

### 2.2. Initial Resistance Characteristics by Sintering Times

**Figure 5:**
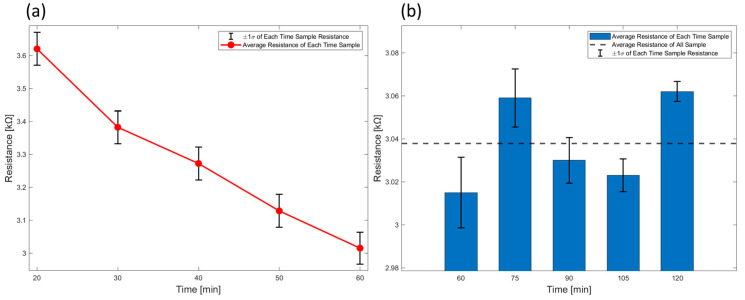
(**a**) Resistance decrease trend caused by sintering for 1 h. (**b**) Variation in resistance measured values between 1 and 2 h of sintering.

A sufficient sintering temperature and duration are required to activate the printed sensor. The sensor must be dried at elevated temperatures to remove the surfactant and form conductive pathways composed solely of activated carbon and graphite particles. The influence of sintering time on the sensors’ initial resistance was quantified using specimens fabricated in a single print batch to minimize batch-to-batch variability. Samples were sintered at 150 °C and resistance was monitored for 2 h. Measurements were acquired at 10 min intervals from 20 to 60 min to capture the pronounced early-time evolution, and at 15 min intervals from 60 to 120 min as shown in Figure 5. This single-batch design, together with the practical constraints of our printing and curing workflow, limited the accessible sintering window to at most 2 h and limited the number of specimens that could be fabricated within the same batch. We explicitly note this limitation so that readers are aware that long-term stability beyond 2 h was not directly verified under identical single-batch conditions. Accordingly, Figure 5 reports the mean response across sensors at each time point, and the error bars represent mean ± σ across samples from the same batch.

Sintering was carried out using a natural convection oven, and resistance was measured using sensors for each sintering time. The resistance of each sensor was measured after it was completely cooled to room temperature. Figure 5a shows the resistance change according to sintering time. The sensor sintered for 20 min exhibited a resistance of approximately 3620.1 Ω, which decreased linearly to 3015.9 Ω after 1 h. Figure 5b shows that after 1 h of sintering, the resistance remained nearly constant. Minor variations were observed, likely due to sensor-to-sensor differences, but no significant resistance change occurred over time. These results suggest that sufficient sintering time affects the dispersion of nanoparticles in the ink.

### 2.3. Differeces of Resistance Change Properties by Printed Layers

**Figure 6:**
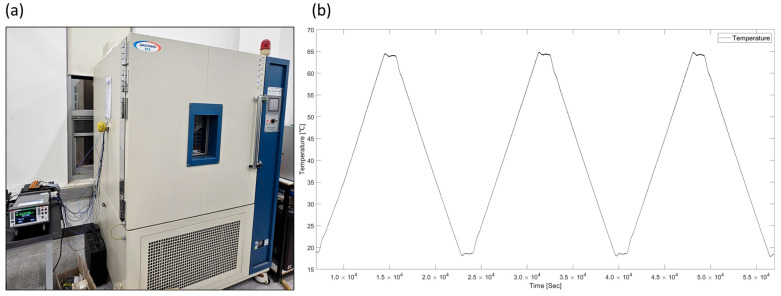
(**a**) Temperature chamber and (**b**) operating temperature cycle.

In EHD inkjet-printed sensors, printing is performed on a microscale. The number of printed layers can also change the sensor’s electrical properties [12]. To evaluate the effects of the sensor’s printed layers, the resistance changes with temperature were tested using a temperature cycle, which referenced the range of temperature data that studied the effects of thermal imbalance on the structure [1]. The temperature cycle using a constant temperature chamber was configured as shown in Figure 6.

**Figure 7:**
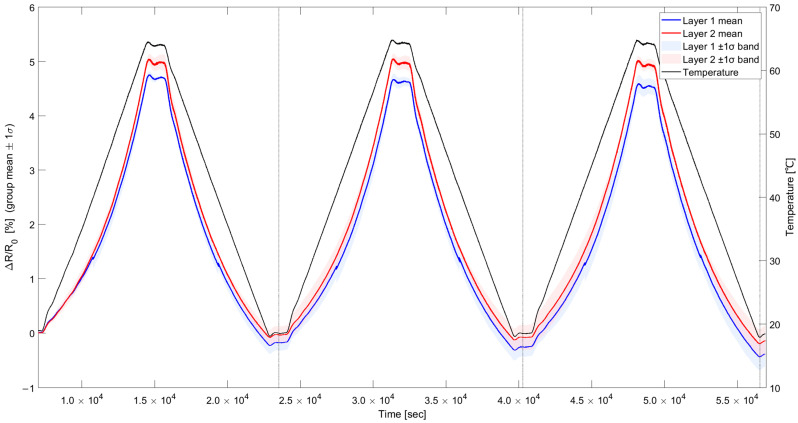
Resistance changes of the sensor depending on the number of printed layers during temperature cycles.

The temperature cycle used in the experiment was set to reach a maximum temperature of 70 °C for 2 h, maintain this temperature for 20 min, decrease to a minimum temperature of 25 °C over the following 2 h, and then maintain the temperature for another 20 min. The actual maximum temperature during the cycle measured using a K-type thermocouple was recorded as 67.5 °C, and the actual minimum temperature was recorded as 19.1 °C. Resistance was measured with a Keithley DAQ6510 using the two-wire DC measurement method. Figure 7 shows the temperature cycle coupled with the resistance change ratio and standard deviation. Within the 20–70 °C range, layer-wise performance of the printed temperature sensor was evaluated using three metrics, namely the mean temperature coefficient of resistance (TCR), the noise-equivalent temperature (NET, computed as the residual standard deviation divided by the slope in °C), and the within-layer standard deviation of the cycle-averaged ΔR/R_0_ shown in Table 3. For the single-layer sample L1, a mean TCR of 0.107–0.108%/°C and a NET of 1.97–2.02 °C were obtained, whereas for the two-layer configuration L2, values of 0.112–0.113%/°C and 1.89–1.99 °C were obtained. These values correspond to a 4.75% increase in sensitivity for L2 and a consistently lower NET, with an average improvement of about 2.05%. In addition, the intra-layer standard deviation of ΔR/R_0_ was lower for L2 than for L1 across cycles, indicating improved sample-to-sample uniformity with stacking. As a result, the research findings demonstrate that the two-layer printed samples exhibit improved sensitivity, temperature resolution, and uniformity compared to the single-layer printed samples.

### 2.4. Analysis of Sensor’s Hysteresis Characteristics

**Figure 8:**
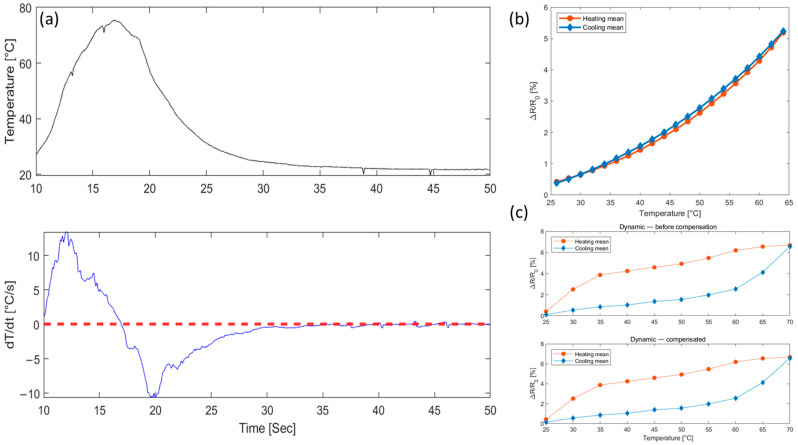
(**a**) Dynamic temperature profile, (**b**) static hysteresis characteristics, and (**c**) dynamic hysteresis and compensation result.

A comprehensive evaluation of the sensor’s hysteresis is a critical prerequisite for accurate temperature calibration. The sensor’s characteristics were evaluated under both slow, static thermal cycles and rapid heating conditions. As summarized in Table 4, negligible hysteresis with a normalized loop area of only 0.077% was observed under static conditions. This is visually confirmed by the nearly overlapping heating and cooling curves in Figure 8b. Conversely, when subjected to the dynamic temperature profile shown in Figure 8a, a pronounced hysteresis loop with a normalized area of 2.604% was measured, as illustrated in the top panel of Figure 8c.

The sensor’s resolution, quantified by the NET, also showed significant degradation in the dynamic test. It is important to note that the NET values presented here are calculated separately for the heating and cooling paths to accurately assess the linearity of each phase. This differs from the cycle-averaged NET in the previous Table 3, where fitting a single linear model to the entire loop caused errors from the heating and cooling paths to partially cancel each other out, resulting in an apparently low NET value. As detailed in Table 4, the more precise path-specific analysis shows the NET increased substantially from approximately 2.053–2.313 °C in static conditions to as high as 14.065 °C and 18.446 °C for the heating and cooling phases in the dynamic test, respectively.

To test whether dynamic hysteresis could be explained by a simple response delay, we applied a first-order lag. As detailed in Table 5 and shown in the bottom panel of Figure 8c, the compensation was ineffective: the loop area remained essentially unchanged, and the NET of both branches showed no meaningful improvement. This indicates that the observed dynamic hysteresis is not attributable to a simple time lag. Instead, it likely arises from electro-thermo-mechanical coupling within the composite, whereby thermal expansion of the polymer binder reconfigures the conductive particle network in a path-dependent manner [31], and thermal-mismatch-induced mechanical stress between the printed ink and the substrate further modulates the electrical response [7,32]. These combined effects produce a complex, rate-dependent error that first-order models cannot capture, underscoring the need for a more sophisticated, data-driven calibration model.

## 3. Calibrations Sensor Resistance Changes According to Deep Learning

### 3.1. Basics of DNN and LSTM Models

The DNN and LSTM models were selected to calibrate the nonlinear resistance change according to the temperature of the sensor. The DNN accepts data from the input layer and performs weighting calculations through the hidden layer. It calculates the predicted value from the output layer, and the process is as shown in Equation (1). $x_{i}$ is the input value, $f\left(x\right)$ is the activation function, $w_{i}$ is value of weight, y is the predicted value after the calculation process, and b is the bias value that introduces nonlinearity.

(1) $$y=f\left(\sum_{i=1}^{n}w_{i}x_{i}+b\right)$$

**Figure 9:**
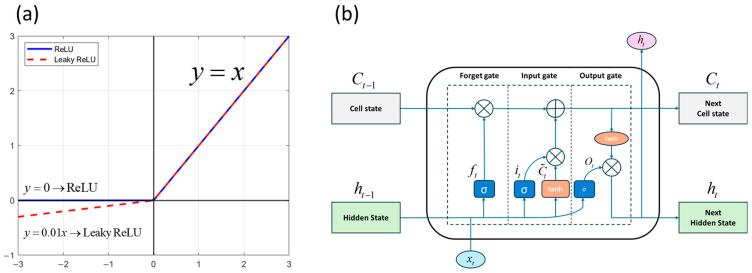
(**a**) ReLU and Leaky ReLU functions and (**b**) LSTM structure.

The activation functions used in the DNN model were Rectified Linear Unit (ReLU) and Leaky ReLU. The ReLU function is defined in Equation (2), and the difference between ReLU and Leaky ReLU is defined in Equation (3). Figure 9a shows the schematic diagram of ReLU and Leaky ReLU.

(2) $$\;f(x)=\max(0,x),\;{\;f}^{\prime }(x)=\left\{\begin{array}{c} 1\;if\;x\geq 0\\ 0\;if\;x<0 \end{array}\right.\to \mathrm{ReLU}$$

(3) Leaky ReLU : f(x)=max(αx,x),   α=0.01

The LSTM model is a modified algorithm of the Recurrent Neural Network (RNN) model that can selectively remember and forget information from previous time steps to address the long-term dependency problem. This functionality is regulated through the forget gate, input gate, and output gate. Figure 9b shows the structure of LSTM model.

The forget gate is defined in Equation (4). $h_{t-1}$ is the hidden state of the previous step, $x_{t}$ is the input value, $b_{f}$ is the bias of the forget gate, σ is the sigmoid function, and $f_{t}$ is the activation value of the forget gate.

(4) $$f_{t}=\sigma \left(W_{f}\cdot \left[h_{t-1},x_{t}\right]+b_{f}\right)$$

The input gate is defined in Equation (5). $i_{t}$ is the activation value of the input gate, ${\tilde{C}}_{t}$ is the candidate cell state value, and $C_{t}$ is the final cell state value. $W_{i},W_{C},b_{i},b_{C}$ are the weight matrices and biases of the input gate and the candidate cell state. The tanh function is defined as the hyperbolic tangent activation function.

(5) $$i_{t}=\sigma \left(W_{i}\cdot \left[h_{t-1},x_{t}\right]+b_{i}\right),\;\;\;{\tilde{C}}_{t}=Tanh\left(W_{C}\cdot \left[h_{t-1},x_{t}\right]+b_{C}\right),\;\;\;{\;C}_{t}=f_{t}\cdot C_{t-1}+i_{t}\cdot {\tilde{C}}_{t}$$

The output gate is defined in Equation (6) using the sigmoid activation function and the tanh activation function to determine how much of the cell state calculated in Equation (5) is to be output. $h_{t}$ is the final hidden state, and $W_{o},\;b_{o}$ denote the weight matrix and bias of the output gate.

(6) $$o_{t}=\sigma \left(W_{o}\cdot \left[h_{t-1},x_{t}\right]+b_{o}\right),\;h_{t}=o_{t}\cdot Tanh\left(C_{t}\right)$$

### 3.2. Preprocess for the Data and Model Performance Evaluation

The data used for learning was normalized and standardized to reduce the effects of the magnitude about temperature and the change in resistance ratio. Normalization was performed using Min-Max scaling in the DNN model, and standardization was performed using Standard scaling in the LSTM model. For each model, the scaling method was selected through parameter studies to minimize prediction error. Min-Max scaling and Standard scaling are defined in Equation (7), where σ denotes the standard deviation.

(7) $$Min-Max\;scaling\;:\;X^{\prime }=\frac{X-X_{min}}{X_{max}-X_{min}},\;\;Standard\;scaling\;:\;X^{\prime }=\frac{X_{i}-\bar{X}}{\sigma }$$

The loss function quantitatively represents the error between the predicted value of the model and the actual value. Both the DNN and LSTM models were trained using the Mean Square Error (MSE), represented by Equation (8) as the loss function. In addition, the Root Mean Square Error (RMSE), the square root of the MSE value, which shares the same unit as the temperature, was defined as the evaluation criterion to directly assess the prediction error. $Y_{i}$ denotes the *i_th_* actual value, $\hat{Y_{i}}$ denotes the *i_th_* predicted value.

(8) $$\mathrm{MSE}:\;\frac{1}{n}\;\sum_{i=1}^{n}{\left(\hat{Y_{i}}-Y_{i}\right)}^{2}\;$$

To evaluate the performance of the trained model, the coefficient of determination (R^2^ score), along with the MSE and the RMSE, were calculated. The R^2^ score, represented by Equation (9), indicates how well the model explains the variability of the dataset. Although the R^2^ score does not directly represent the absolute error of the model, it provides insight into the model’s ability to explain variance in the target variable.

(9) $$R^{2}\;:\;1-\frac{\sum {\left(Y_{i}-{\hat{Y}}_{i}\right)}^{2}}{\sum {\left(Y_{i}-\bar{Y}\right)}^{2}}\;$$

### 3.3. Dataset for Model Training and the Structure of DNN and LSTM

In this section, the architecture of the machine learning models and the structure of the training data are described. Dynamic temperature variation data from Section 2.4 are excluded from the training set. Only the static temperature cycle data introduced in Section 2.3 were used for training. Evaluation of each model using dynamic temperature variation data will be presented in Section 4: Results and Discussions.

**Figure 10:**
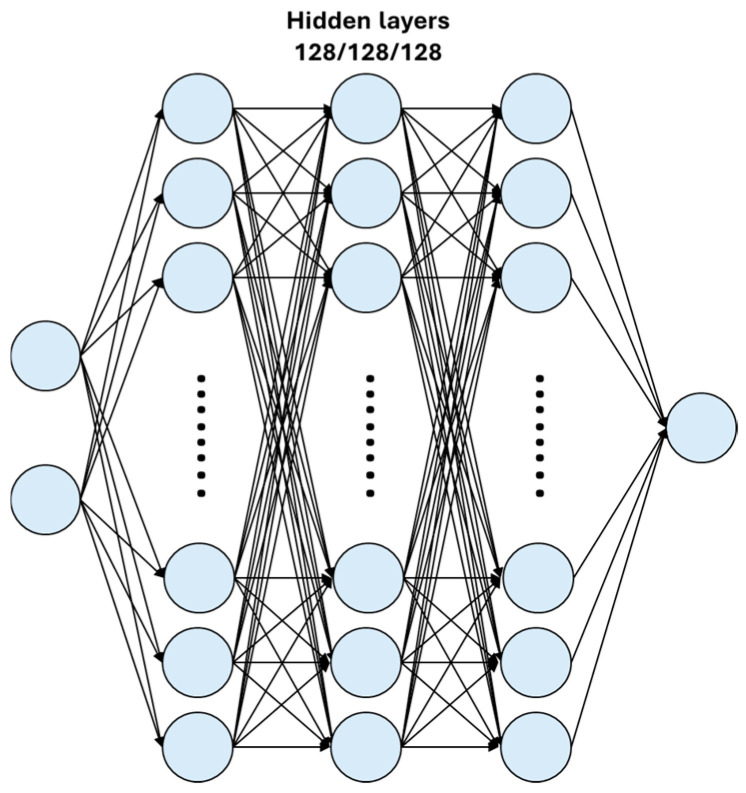
Schematic diagram of DNN structure.

The DNN model developed for temperature calibration consisted of three hidden layers with a 128/128/128 neuron configuration. Activation functions for the hidden layers were compared using both ReLU and Leaky ReLU. Figure 10 shows the structure of DNN model. The LSTM model was compared by training a model with two LSTM layers of 32/32 structure and the hidden layer structure used in DNN, and a model with a simplified structure of one LSTM layer and one hidden layer to reduce model complexity to reduce overfitting.

**Figure 11:**
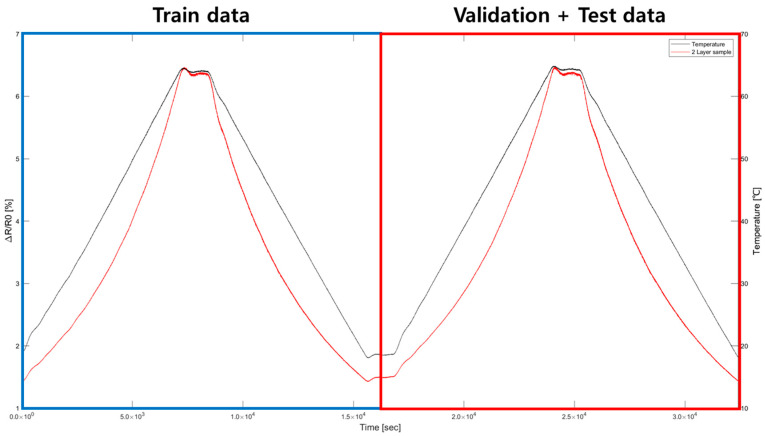
Temperature cycle data for modeling DNN and LSTM algorithms.

The data used for model training was obtained from two cycles, as shown in Figure 11, from the cycles measured in the temperature chamber. The dataset used for training, validation, and testing was divided into 50%, 25%, and 25%, respectively, as shown in Table 6, to create the model. A total of 94,580 data points were collected, with the first cycle used for training using a total of 47,290 data points. The validation and test data were trained to have generality by using the latter cycle: the 47,920 data points of the latter cycle were divided in half to conduct the validation and test processes.

The input features included the resistance values and resistance change ratios, while the target output was the corresponding temperature, accurately measured using a K-type thermocouple. In the case of the LSTM model, the time step was set to 100 to reflect the time-series data. Both the DNN model and the LSTM model were made into a structure that outputs resistance and resistance change ratio as input data, and calibrated temperature as output data. Model performance was evaluated by comparing the predicted temperature value through a polynomial regression equation with trained models.

## 4. Results and Discussions

This section presents and discusses the performance of the machine learning models developed for temperature calibration. Section 4.1 first details the hyperparameter optimization process and evaluates the fundamental performance of the models on the quasi-static data used for training, comparing them against a conventional baseline. Next, Section 4.2 presents the critical test of the models’ ability to generalize to unseen, dynamic conditions characterized by severe hysteresis. Finally, Section 4.3 provides a concluding discussion on the practical characteristics of the machine learning approach and analyzes external sources of error, including the effects of humidity and data imbalance.

### 4.1. Model Optimization and Performance on Static Temperature Data

During training, hyperparameters appropriate to the data were selected and their effects were interpreted, a step that is essential for extracting insight from data with machine learning. The condition of the data was also recognized as a major factor affecting model outputs and therefore required careful attention. In this section, the effects of each model’s hyperparameters on the training outcomes are first explained, and the influence of the data condition on learning is then discussed.

The number of epochs used for training the DNN model was 100, while the LSTM model was trained using both 100 and 300 epochs. The batch size was fixed at 32. The optimizer used for both models was Adaptive Moment Estimation (Adam), a widely adopted method for training deep neural networks. Although the model included multiple hidden layers, which could improve performance in predicting nonlinear relationships, this increased complexity may also lead to overfitting. To solve this problem, an early stopping callback function was used to terminate the learning early at the point when the optimal weights were found. Models with early stopping were compared to those trained without the early stopping condition.

**Figure 12:**
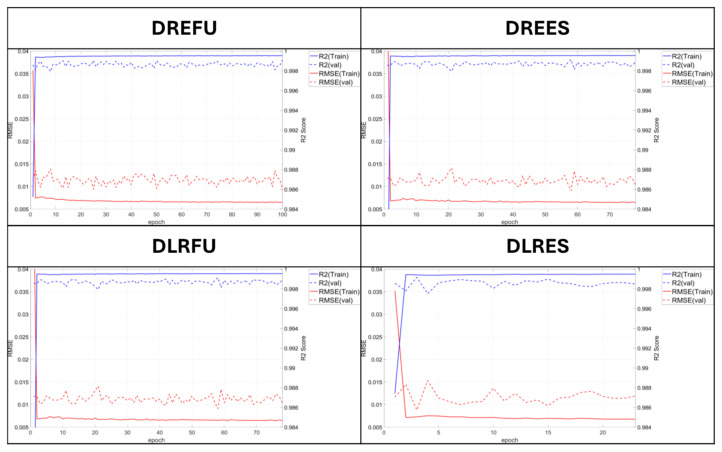
Changes in R^2^ score and RMSE values by epoch progression [DNN models].

**Figure 13:**
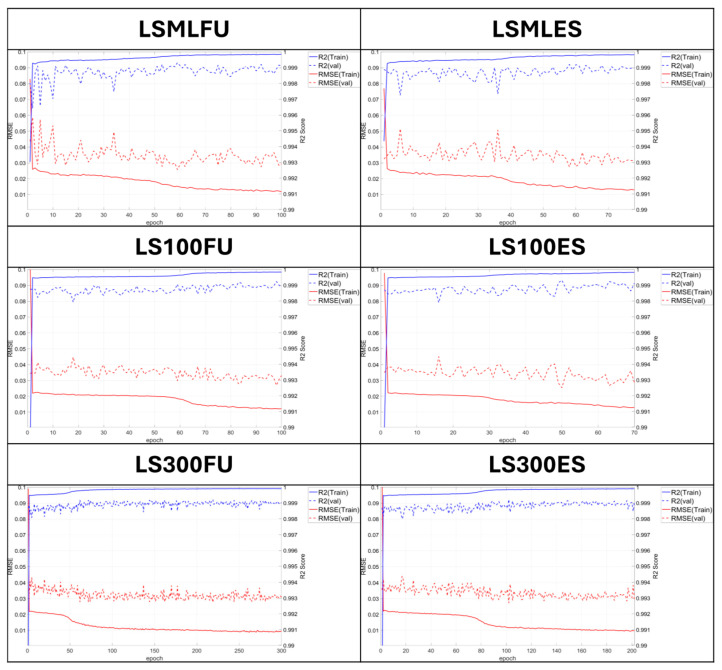
Changes in R^2^ score and RMSE values by epoch progression [LSTM models].

For DNN models, early stopping was configured to stop the training if the validation loss did not decrease for 20 epochs. For LSTM models, early stopping conditions were configured to stop the training if the validation loss did not decrease for 20 and 100 epochs, depending on the model’s structure. For the model trained on 300 epochs, the early stopping threshold was set to 100 epochs, considering its relatively shallow structure and extended training duration. Table 7 and Table 8 show the activation functions, number of dense layers, early stopping status, and total epochs used in each model. Figure 12 and Figure 13 show the R^2^ scores and RMSE values for the DNN and LSTM models, respectively, based on parameter configurations summarized in Table 7 and Table 8.

As shown in Table 7, abbreviations were used to define the models to represent the characteristics of each model. For models trained using DNN, the prefix “D” was assigned to distinguish them from LSTM-based models. Models employing ReLU and Leaky ReLU as activation functions were labeled “RE” and “LR”, respectively. “FU” denotes a model trained for the full number of epochs, whereas “ES” indicates a model to which early stopping was applied during training. For the LSTM model, as shown in Table 8, the configuration with two LSTM layers and three hidden layers was denoted as LS-ML (LSTM–Max Layer). As previously described, the model variants were labeled LSML-ES and LSML-FU, depending on whether early stopping was applied. For shallow LSTM models, an alternative abbreviation rule was adopted, where the total number of training epochs (100 or 300) replaced “ML” to indicate the model type and training condition.

During the training of both models, the training RMSE exhibited an accelerated decreasing trend after 40 epochs, whereas the validation RMSE showed no significant variation. Considering the possibility of overfitting during training, the model depth was reduced, and a parameter study was conducted under a fixed number of epochs. When a model with a reduced architecture, consisting of one LSTM layer and one hidden layer, was trained, it was compared to models with and without early stopping under the condition of 100 training epochs. Based on the comparison of RMSE values and R^2^ scores, the model trained without early stopping showed lower prediction error and better performance in both metrics. In addition, compared to the existing model configured with two LSTM layers and three hidden layers, both simplified models exhibited improved calibration performance, indicating a likelihood of overfitting in the original configuration and reduced generalization in the validation and test phases.

The observed improvement in performance with increasing epochs in the shallow LSTM model suggests insufficient iterative training. The number of epochs was increased to 300 to identify the optimal model configuration. The early stopping condition was configured to terminate training if no further reduction in validation loss was observed over 100 consecutive epochs, under the assumption of extended training duration. As a result of model training, both the models without early stopping and those with early stopping exhibited lower RMSE values and higher R^2^ scores compared to the previously trained deep-layered LSTM model and the shallow-layered model trained for 100 epochs. An examination of RMSE and R^2^ values at each epoch indicated that the shallow-layered model began to demonstrate superior calibration performance after approximately 80 epochs, with performance plateauing beyond 200 epochs. The performance metrics of each model are summarized in Table 9 and Table 10, respectively.

**Figure 14:**
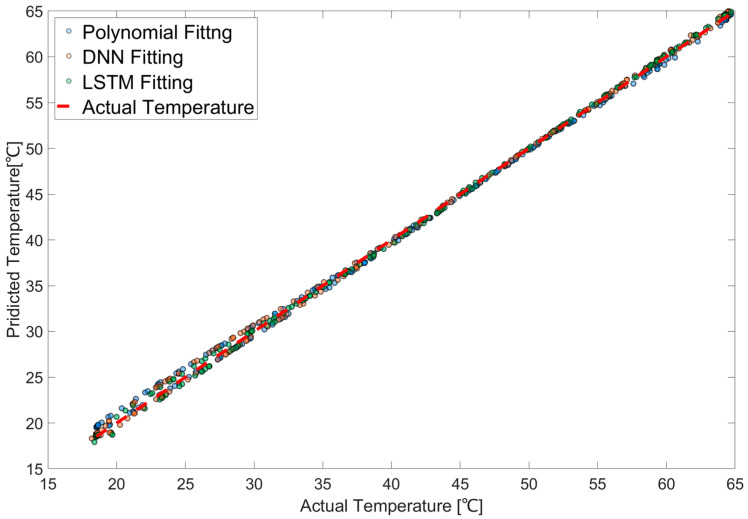
Scatter plot of predicted temperature samples from each model and actual temperature.

To evaluate the distribution of the predicted temperature data, a scatter plot was constructed using the calibration results obtained from the polynomial regression shown in Figure 14, along with the outputs of the DNN and LSTM models that yielded the lowest prediction errors. The polynomial regression equation is shown in Equation (10), where variable a is the scaled resistance change rate and b is the scaled resistance value. A polynomial regression with L1 regularization was employed. Inputs were expanded to polynomial terms, and all features were standardized to zero mean and unit variance so that the penalty acted uniformly across coefficients. The polynomial degree was selected by grid search over degrees two through five using time-ordered cross-validation that preserved temporal order and applied an embargo around fold boundaries to prevent leakage. The regularization strength was selected in the same procedure. The final specification was refit on the full training split with the selected hyperparameters, and generalization was assessed on a held-out test split. Agreement among training errors, cross-validation errors, and test errors indicated that overfitting was effectively controlled. The L1 penalty shrank unsupported coefficients to zero, which reduced effective model complexity, suppressed variance inflation from higher order terms, and yielded a parsimonious and stable regression equation. The data was confirmed for a total of 600 data distributions by random sampling of 200 points from each of the three calibrated models. The models were compared using scatter plots of the DLRES and LS300ES results. Quantitative analysis showed that the DNN model produced a distribution closer to the ideal diagonal line than the polynomial model, while the LSTM model exhibited a distribution closer to the ideal than the DNN model.

(10) $$\hat{y}=-42.053+15.461a-17.020a^{2}+15.561ab+0.581b^{2}+17.526a^{3}-31.242a^{2}b+12.508ab^{2}+0.989b^{3}$$

### 4.2. Generalization to Dynamic Hysteresis Conditions

While Section 4.1 established the models’ superior performance under the quasi-static conditions used for training, the true test of their utility for real-world applications lies in their ability to generalize to unseen, dynamic conditions. Therefore, to verify their performance against severe hysteresis, the previously trained models were evaluated using the rapid temperature variation data described in Section 2.4. As summarized in Table 11, a stark contrast in performance was observed. The conventional Lasso–Polynomial model completely failed, yielding a negative R^2^ score of −0.441 and an RMSE of 12.451 °C. In contrast, the DNN model outperformed polynomial regression with a significantly lower RMSE of 9.309 °C, and the LSTM model delivered the best predictive performance, achieving an R^2^ score of 0.799 and an RMSE of 4.899 °C.

Importantly, this significant improvement was obtained even though the hysteretic behavior associated with rapid temperature changes was not included during the training phase. This result highlights a critical difference in generalization capability. The LSTM model, by learning the underlying temporal dynamics of the sensor’s response, successfully generalized beyond its training regime to provide an effective remedy for hysteresis-induced errors. In contrast, conventional polynomial regression, as a static curve-fitting method, fundamentally lacked the ability to generalize to the dynamic conditions. For future work, it is anticipated that by including the rate of temperature change as an input feature and incorporating the dynamic case data into the training set, a more robust and accurate calibration model capable of successfully predicting temperatures across all static and dynamic conditions can be developed.

### 4.3. Analysis of Model Characteristics and Error Sources

In this study, a flexible temperature sensor was fabricated using EHD inkjet printing to effectively measure thermal imbalance. Performance evaluation based on resistance changes with respect to temperature revealed a nonlinear response, which was addressed by performing temperature calibration using DNN and LSTM algorithms and comparing their performance against a polynomial regression baseline. When analyzing the characteristics of the machine learning models, both DNN and LSTM have distinct advantages and trade-offs.

The advantage of calibrating temperature using DNN is that performance enhancement can be achieved through optimization of the density of the neural network and the activation function. In this study, the activation functions were divided into ReLU and Leaky ReLU, and the respective models were compared. Also, the temperature calibration performance of the model was improved by applying early stopping. However, the method of calibrating the temperature of the sensor through DNN requires more computing resources than the method of correcting errors through regression equations, and suboptimal hyperparameters can cause overfitting, resulting in lower performance than regression equation models. The DNN calibration model designed in this study prevented overfitting by performing early stopping, and it was confirmed that the RMSE value was improved by up to 20.603% compared to the polynomial regression equation.

The advantage of calibrating temperature using LSTM is its ability to process time-series data, retain previously trained information, and incorporate temporal dependencies into the learning process. This characteristic makes LSTM particularly suitable for sensors, such as temperature sensors, that require long-term monitoring of data variation. However, there is a limitation that it may be difficult to apply if time-series data cannot be used when continuous data measurement is not possible. Furthermore, LSTM requires longer training time than conventional DNN models because time-series data is also trained together. By employing early stopping and simplifying the architecture, the proposed LSTM calibration model reduced the RMSE from 0.5077 for the polynomial baseline to 0.3373, representing a 33.563% reduction in the RMSE value.

**Figure 15:**
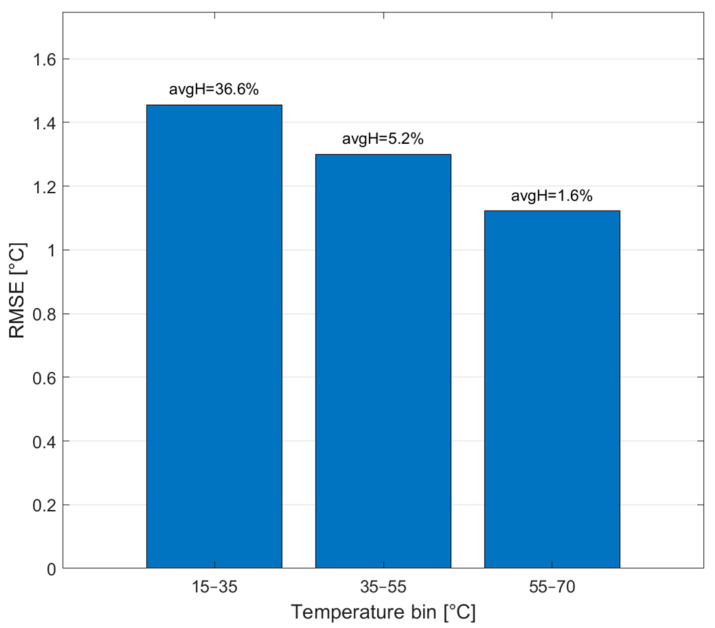
Effect of temperature and humidity on model error. The highest RMSE was observed in the 15–35 °C bin, consistent with the highest mean humidity.

Although the machine learning models demonstrated superior overall performance, a detailed analysis of the prediction errors revealed several contributing factors. For instance, greater errors were observed in the room-temperature range than in the high-temperature range. This behavior was attributed to increased humidity caused by condensation in the chamber during repeated thermal cycles, which adversely affected the sensor. To quantify this effect, humidity was recorded with a DHT21 sensor, and the operating range was divided into three temperature ranges, 15–35 °C, 35–55 °C, and 55–70 °C. As shown in Figure 15, the results confirmed that lower temperature and higher humidity were associated with larger errors. To address this issue, future work should consider sensor coating and additional environmental testing.

**Figure 16:**
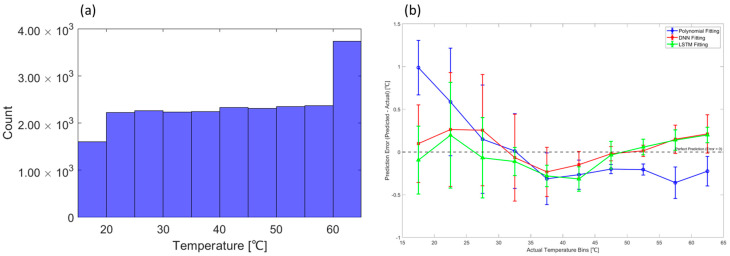
(**a**) Temperature data used in validation and test. (**b**) Error bar graph of calibration values by temperature range.

In addition to environmental factors such as humidity, the distribution of the training data itself was found to influence the learning results [33]. To investigate this effect, the distributions of the validation and test data were analyzed. As shown in the histogram in Figure 16a, the amount of data in the under 20 °C range is small compared to other ranges. Conversely, the range of 60 °C or more, where the error and its standard deviation start to increase again, has the most data, as seen in Figure 16b. This phenomenon was caused by the section that maintains a constant temperature during the cycle, suggesting that the unbalanced distribution of data affects learning. To mitigate these calibration errors, future work will focus on improving the data distribution within the temperature cycle.

**Figure 17:**
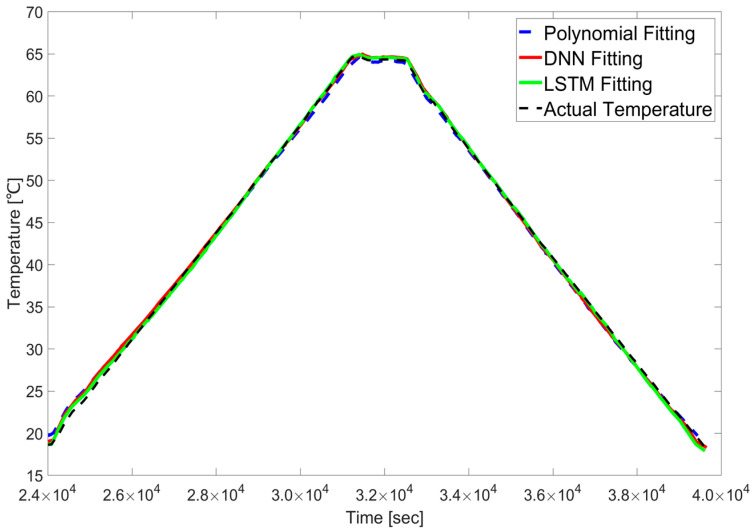
Actual temperature and predicted temperature results by each model.

Despite these identified sources of error, a final visualization of the entire temperature cycle confirmed the overall superiority of the machine learning models. As illustrated in Figure 17, the calibrated results from the DNN and LSTM models demonstrated a closer alignment with the overall temperature trend compared to those derived from the polynomial regression method, even with the discrepancies in regions with unbalanced data and humidity effects.

## 5. Conclusions

In this study, a flexible temperature sensor was fabricated using EHD inkjet printing to address the challenge of temperature monitoring in structural health applications. The primary finding was that while the sensor’s static nonlinearity can be calibrated effectively, its reliability is critically challenged by a complex dynamic hysteresis that emerges under rapid thermal changes. Our analysis showed the limitations of conventional methods, including first-order lag compensation and polynomial regression, in adequately compensating for this severe path-dependent error, where the sensor’s output depends not just on the current temperature but also on its prior thermal history.

To address this challenge, DNN and LSTM models were developed, and their calibration performance was compared against a conventional polynomial regression. Under quasi-static conditions, all approaches proved effective in estimating the temperature. However, the critical differentiator was the models’ ability to generalize to unseen, dynamic conditions.

In this more challenging regime, the LSTM model successfully generalized where the conventional method failed, delivering a predictive RMSE of 4.90 °C, which was vastly superior to the 12.45 °C RMSE of the polynomial regression. This finding demonstrates that while simple calibration is sufficient for static environments, a data-driven approach like LSTM, capable of learning the underlying temporal dynamics, is essential for ensuring reliability in real-world applications where both static and dynamic conditions occur.

While this study confirms the potential of the proposed method, future work is needed. The analysis on error sources indicated that performance could be further enhanced by mitigating the effects of humidity and data imbalance. Furthermore, a more robust model capable of successfully predicting temperatures across all static and dynamic conditions could be developed by incorporating the rate of temperature change as an input feature and including dynamic data in the training set.

## Figures and Tables

**Table 1:** Average line width and standard deviation by printing speed parameters.

Printing Speed [mm/s]	Average Line Width [μm]	STD of Line Width [μm]
100	227.905	40.014
150	194.258	31.908
200	171.500	31.282
250	161.917	21.147
300	134.793	27.542
350	126.329	28.752
400	122.213	13.211
450	111.955	15.631
500	102.520	19.366
550	106.382	17.277
600	100.367	12.141
650	101.443	14.434
700	98.214	16.020
750	102.963	11.233
800	99.733	13.358
850	100.177	15.366
900	102.773	12.790
950	105.686	14.728
1000	101.760	16.996

**Table 2:** Printing parameters of carbon ink for EHD inkjet printing.

Parameter	Value [Unit]
Move acceleration [X, Y axis]	250 [mm/s^2^]
Print speed [X, Y axis]	250 [mm/s]
Printing position [Z axis]	1000 [μm]
Printing voltage [DC]	2.3 [kV]

**Table 3:** Evaluation index of sensor resistance changes according to temperature cycles.

	L1 STD [%]	L1 TCR [/°C]	L1 NET [°C]	L2 STD [%]	L2 TCR [/°C]	L2 NET [°C]	
Cycle 1	0.105	0.107	1.970	0.0226	0.112	1.892	
Cycle 2	0.171	0.108	2.015	0.0801	0.113	1.986	
Cycle 3	0.218	0.108	1.984	0.1223	0.113	1.971	

**Table 4:** Hysteresis performance comparison at each thermal condition.

	Static Thermal Condition	Dynamic Thermal Condition
NET_Heating [°C]	2.313	14.065
NET_Cooling [°C]	2.053	18.446
Hysteresis_Area [%]	0.077	2.604

**Table 5:** Effect of first-order compensation on dynamic thermal data.

	Before Compensation	After Compensation
NET_Heating [°C]	14.065	14.059
NET_Cooling [°C]	18.446	18.457
Hysteresis_Area [%]	2.604	2.605

**Table 6:** Split training, validation, and test data for model learning.

Total Data	Train Data	Validation Data	Test Data	Input Data	Output Data
94,580 [100%]	47,290 [50%]	23,645 [25%]	23,645 [25%]	Resistance, Resistance ratio	Temperature

**Table 7:** Model properties of DNN and LSTM models.

Model	Activation Function	Epoch	Early Stopping	Layers
DREFU	ReLU	100	None	128/128/128 (Hidden layers)
DREES		78	O	
DLRFU	Leaky ReLU	100	None	
DLRES		23	O	

**Table 8:** Model properties of LSTM models.

Model	Time Step	Epoch	Early Stopping	Layers
LSMLFU	100	100	None	32/32 (LSTM layers) 128/128/128 (Hidden layers)
LSMLES		78	O	
LS100FU		100	None	16 (LSTM layer) 128 (Hidden layer)
LS100ES		70	O	
LS300FU		300	None	
LS300ES		202	O	

**Table 9:** Prediction results of the polynomial regression and DNN models.

Model	RMSE [°C]	R^2^ Score
Lasso—Poly	0.5077	0.9988
DREFU	0.4602	0.9990
DREES	0.4140	0.9992
DLRFU	0.4336	0.9991
DLRES	0.4031	0.9992

**Table 10:** Prediction results of LSTM models.

Model	RMSE [°C]	R^2^ Score
LSMLFU	0.4279	0.9991
LSMLES	0.4126	0.9992
LS100FU	0.3616	0.9994
LS100ES	0.3792	0.9993
LS300FU	0.3680	0.9994
LS300ES	0.3373	0.9995

**Table 11:** Performance summary for models tested under rapid temperature change.

Model	R^2^ Score	RMSE [°C]	MAE [°C]
LSTM	0.7989	4.8987	4.0880
DNN	0.4199	9.3089	7.0781
Lasso-Poly	−0.4407	12.4510	10.2030

## Data Availability

The data is available upon request from the corresponding authors.
